# Effects of lemon decoction on malaria parasite clearance and selected hematological parameters in *Plasmodium berghei* ANKA infected mice

**DOI:** 10.1186/s12906-020-2820-1

**Published:** 2020-01-30

**Authors:** Kelvin M. Shija, Ramadhani S. O. Nondo, Doreen Mloka, Raphael Z. Sangeda, George M. Bwire

**Affiliations:** 1 Department of Pharmaceutical Microbiology, School of Pharmacy, Muhimbili University of Health and Allied Sciences, P.O. Box 65013, Dar es salaam, Tanzania; 2 Department of Biological and Pre-Clinical Studies, Institute of Traditional Medicine, Muhimbili University of Health and Allied Sciences, P.O. Box 65001, Dar es Salaam, Tanzania

**Keywords:** *Citrus limon*, *Plasmodium berghei*, Antimalarial activity, Mice

## Abstract

**Background:**

Citrus plants particularly lemon (*Citrus limon* L.) concoctions are ethno-medically used for treatment of infectious diseases including malaria. Therefore, we set an experiment to investigate the effects of lemon decoction in mice infected with *Plasmodium berghei* ANKA parasites*.*

**Methods:**

Antimalarial activity was determined using Rane’s curative test on 25 Theiler’s albino mice. Twenty mice were each injected with 2 × 10^7^ infected red blood cells (iRBCs). The mice were divided into four groups, consisting of five mice per group. Each group received an oral dose of either 5% carboxymethyl cellulose/placebo (negative infected control), lemon decoction (*Citrus limon* [CILI extract]) alone or a combination of artemether/lumefantrine (A/LU, 28 mg/kg) and CILI extract and A/LU alone. A fifth group of mice consisted of uninfected mice as parasite-negative control.

**Results:**

Within 72 hours after initiation of treatment, the mean percentage parasitemia ± standard deviation of the CILI extract group (24.2% ± 9.83%) was lower compared to placebo group (40.0% ± 14.78%), *p* = 0.037. CILI extract group was found to have an increased survival rate (11 days ± 1.6 days) as compared to placebo group (8.6 days ± 3.4 days), *p* = 0.226. Mice in the combination group (A/LU + CILI extract) had the highest mean counts in terms of hemato-immunological parameters, whereas those in the CILI extract alone had the lowest hematocrit levels. The study also found that mice that received a combination of CILI extract and A/LU exhibited a decreased lag time with regards to time required to clear 99% of parasites (58.8 h vs. 64.2 h, *p* = 0.681) as compared to the A/LU alone group.

**Conclusion:**

Lemon decoction demonstrated antimalarial activity in mice infected with *P. berghei* ANKA through parasites suppression by 39% as compared to those received placebo. However, when used alone, lemons did not suffice as a cure but in combination with standard antimalarials, lemons promoted early parasite clearance with an improved hematological parameters.

## Background

Malaria in rodents is caused by parasites of *Plasmodium* species. *P. yoelii, P. chabaudi, P. vinckei* and *P. berghei* [1], whilst the species *P. malariae*, *P. vivax*, *P. falciparum, P. knowlesi* and *P. ovale* affect humans. It is estimated that 90% of malaria cases occur in sub-Saharan Africa and the groups most affected are pregnant women and children [2]. Nearly half of the world’s population is at risk of malaria and its severe cases result in death as a result of gross anemia if not treated in time [3, 4].

The treatment failure due to artemisinin-based combination therapies (ACTs) has been attributed to its uneven pharmacokinetics among individuals and its requirement to be taken with a fatty meal to improve absorption [5, 6]. Moreover, the dosage, the compliance and the immune status of the patient are all implicated [7]. Failure may also happen in immune-compromised patients as they are likely to be taking other medications like efavirenz. Therefore, this may affect their pharmacokinetics when taken together with ACTs [8].

To overcome these problems, new prevention and treatment options are therefore needed. Be it new agents or agents that can help boost other mechanisms to curb malaria infections, including via different immune-modulated approaches are both welcome initiative. The use of traditional medicines emanates from ancient history, as evidenced by historical inscriptions like the Materia Medica and the Medical papyri. The discovery of modern medications used in the treatment of malaria and various other diseases has its roots in traditional medicine. For instance, quinine is a pure extract obtained from the bark of *Cinchona spp,* which was used to treat malaria traditionally [9, 10].

The ACTs were developed from *Artemisia annua*, which is used in the treatment of malaria in Chinese traditional medicine. *Artemisia annua* (q*l̄* nghāo in Chinese) is a medicinal herb with a sesquiterpene active ingredient isolated and analysed chemically in detail in l972. A combination with lumefantrine gave rise to artemether/lumefantrine (A/LU) drugs. The combinations are currently recommended by the World Health Organization (WHO) as the first-line drugs for the treatment of uncomplicated malaria [11].

Citrus plants are ethno-medically used for infectious, non-communicable diseases and general well-being. Studies show that lemon (*Citrus limon* L.) concoctions are used by traditional folks to treat malaria [12] and limes used together with ACT (A/LU) promoted rapid parasite clearance and prevented treatment failure [13]. Besides, positive hematological (cell-mediated and humoral) effects of lemons were reported in growing rabbits [14]. However, some societal prejudices and beliefs link lemons to cause anemia, while some attribute them to an immune-boosting effect, which is also supported by some studies [14]. It must be emphasized, through literature review, it has been found that; low levels of hemoglobin and iron protect the erythrocytes from malaria parasites [15].

The detailed description of the health effects of the various citrus plant, especially lime and lemon [13–16] plus the pieces of evidence generated from non-lemon antimalarial activities in rodent animal models [10, 12], are intriguing. We, consequently, found it imperative to carry out this study to establish the effects of lemon decoction using mice model.

## Methods

### Collection and preparation of lemon decoction

The fruits were collected from Msigani village, located in Ubungo District, Dar es Salaam region, Tanzania. Lemons were collected during the intermittent season of March 2018. Mr. A botanist from the University of Dar es Salaam, Tanzania, authenticated the lemon tree and prepared the voucher specimen with collection number KS1. All specimen vouchers were stored in the Herbarium of the Institute of Traditional Medicine (ITM), Muhimbili University of Health and Allied Sciences (MUHAS).

Mature lemons weighing 142 ± 8.2 g were harvested directly from lemon tree ensuring their freshness. Harvested lemons were then stored in a refrigerator at 4 °C in the laboratory at ITM within 12 h of plucking. The method of preparation of the decoction in the laboratory mimics how the remedy is prepared traditionally; “...to make the concoction 3 mature lemon (*Citrus limon*) fruit are cleaned to remove any dust they may contain. They are each cut into four pieces. The 12 lemon fruit pieces were put into 1¼ litres of water and boiled for 12 minutes, then the mixture is left to cool. When cool, the juice is separated from the solid material. A dose from the cold lemon juice is made by measuring out a glassful (which was taken to be about 175-200 mL, ≈188mL). To that a tablespoonful of raw honey is added, stirred, and when well mixed; that glassful is taken by the sick. This is repeated three times a day. Afresh mixture will normally have to be prepared each day” [17]. Lemon fruit decoction was freshly prepared in the laboratory on each treatment day. Preparation involved chopping a single lemon into small pieces then placing the pieces in 208 mL of distilled water in 500 mL, flat-bottomed glass bottle covered with aluminium-foil. The flasks where then heated to boil at 100 °C for 45 min. Then the decoction was then allowed to cool to room temperature (about 24 °C). Cooling was aided by running a stream of lukewarm water over the closed glass bottle containing the lemon decoction. Then the juice from the boiled lemons was decanted and filtered with cotton gauze ready for use.

### Standard drug preparation of artemether/lumefantrine (a/LU)

A/LU (Lumartem®, CIPLA Ltd., Pithampur, India) was purchased from a local shop in Dar es Salaam. One tablet weighing 140 mg was suspended in 40 mL 0.5% carboxymethyl cellulose (CMC) to make a stock solution of 0.35 mg per 0.1 mL. An equivalent human therapeutic dosage was prepared and used corresponding to in mice (28 mg/kg /day per mouse) that is about 4 mg/kg artemether and 24 mg/kg lumefantrine [18, 19].

### Animals

Young adult male and female Theiller’s white albino mice (*Mus musculus*) aged about three to four weeks weighing 28.00 ± 1.72 g were collected from the animal house of the Institute of Traditional medicine and maintained in the laboratory. They were acclimatized to the laboratory conditions for five days at 12 h light/12 h dark cycle and a room temperature of 22–27 °C. All animals were housed in polycarbonate cages and provided witfood (Hill broiler finisher pellets, Hill®, Dar es Salaam, Tanzania) and water ad libitum. General hygiene, such as cleaning the cages was done routinely. To minimize discomfort, injection of parasites and collection of blood for counting parasites was done via tail veins while lemon decoction and A/LU suspension were given orally. All animals that died because of malaria were disposed by incineration.

### Malaria parasites

*Plasmodium berghei* ANKA (MRA-311) strain parasites were obtained from BEI-Resources/ATCC® (Manassas, Virginia**,** USA). The parasites were received frozen in dry ice and then transported to the laboratory where they were thawed at room temperature before injected to the mice via intraperitoneal route. The parasites were maintained in mice by continuous re-infection of naïve mice.

### Euthanasia

Exsanguination method was used in mice that received standard treatment, this was also part of the terminal blood collection procedure. Mice were anaesthetized in accordance with AVMA Guidelines for the Euthanasia of Animals: 2013 Edition. Unconsciousness was confirmed by lack of movement even with pinching of tail/leg. From the mice blood samples were then immediately drawn with one mL syringes moistened with heparin by cardiac puncture procedure. The amount of blood needed could not allow for the survival of mice. All mice died of exsanguination and were then disposed off by incineration.

### In vivo antimalarial test

#### Infection of mice

The parasitemia of the donor mouse was determined before the animal anesthetized. This was followed by a cardiac puncture to collect donor blood in a heparinized tube. Immediately after, the blood was then diluted using normal saline (0.9% NaCl) to obtain a standard inoculum (1 × 10^8^). A standard inoculum of 0.2 mL of infected blood equivalent to 2 X 10^7^ *P. berghei-*infected erythrocytes was then inoculated via tail vein to the test mice.

#### Rane’s test

Evaluation of the therapeutic potential of CILI decoction was done as per the method described by Ryley and Peters [1]. After acclimatization for five days, on day 0, mice were inoculated via tail vein with 2 × 10^7^ infected red blood cells and then kept for 72 h without treatment. Three days post-infection (72 h) initial levels of parasitemia were determined and then randomized into groups of five mice. Care was taken to ensure mice in all cages had approximately the same level of parasitemia. Each cage had a single male and four female mice. Treatment labels were then randomly assigned to each cage and then treated accordingly. Mice in the CILI extract group were fed with 8 mL/ kg body weight, twice a day. This was done to double the dose of the decoction as compared of traditional preparation calculated to be about 4.7 mL/kg body weight thrice a day. This strategy also allowed for some flexibility in the dosing frequency to become twice a day instead of three times a day in the conventional remedy, hence minimizing discomfort to the animals too [17]. The mice in A/LU alone group received a suspension of 4 mL A/LU/ kg bodyweight,twice a day orally (28 mg Artemether/lumefantrine /kg/day). Whereas the combination group received 8 mL / kg bodyweight (4 mL A/LU, 4 mL CILI) orally twice a day. The group of mice that acted as the negative control mice received 8 mL./ kg bodyweight of 0.5%CMC distilled water. Treatment was in the order of; Negative controls, CILI extract, then combination (starting with CILI extract then A/LU). The dosing/feeding was done twice daily (at 9–10 am and 9–10 pm) for four [4] days. An additional group of five mice were uninfected and acted as parasite-negative control. Blood samples for thin smear giemsa staining were prepared from the tail blood of each mouse at intervals of 0, 6,12 and 24 for a duration of five days. This was done to monitor parasitemia, parasite clearance rates and estimation of lag phase.

#### Determination of parasitemia

Thin blood smears were prepared on 76 mm X 25.4 mm glass microscope slides (Yancheng Hongda Medical Instruments Co Ltd., Jiangsu, Baocai, China). The fixing was done in absolute methanol for 15 s then stained with Giemsa stain at pH 7.2 for 20 min. The stained slides were washed with distilled water and then air-dried at room temperature. The stained slides were examined under the light microscope (OLYMPUS CX31RBSF (Olympus Optical Co Ltd., Philippines) using oil immersion at 100x magnification objective. Images of the slides under the microscope were taken and digitally processed then counted with the aid of Cell Count Aid software (Victorian Bioinformatics Consortium, Victoria, Australia). Each slide was examined at several fields to count the total number of infected RBC versus total RBCs in the field. A minimum of 1000 total RBCs were counted per slide and the percentage parasitemia was determined using the formula;

$$ \% Parasitemia=\frac{Number\ of\ parasitized\  RBC}{Total\ number\ of\  RBC}\times 100 $$

Values of percentage parasitemia were used to calculate percentage suppression using the formula:

$$ \% Suppresion=\frac{\left( mean\ parasitemia\ of\  neg.- parasitemia\ of\ test\right)}{mean\ parasitemia\ of\ negative.}\times 100 $$

#### Anti-plasmodial effect of the lemon decoction, the sure cure potential (SCP) of the decoction

The sure cure potential formula was used to illustrate the therapeutic potential further, parasite killing potential and the absolute effectiveness of treatment. This mathematical technique was applied to each treatment group;

$$ Sure\ curative\ potential=\left(1-\frac{Po-\left( Pt- Po\right)\Big)}{Po}\right)x\left(\pm \right)100 $$

Where;

Po: Initial value of a tested parameter (in this case, parasitemia before the commencement of treatment). Po varies depending on initial parasitemia of a particular group.

Pt: Final value after treatment (in this case final parasitemia levels after treatment).

±: Multiplied as per the desired outcome (In this case it is negative (−).

Applicability of this equation can be extended to other studies where Po can be replaced with normal range. It forms a means to compare outcomes, even when the control group is unreliable or missing altogether. The output (answers) calculated this way paints a clear picture of efficacy as opposed to relative outputs of suppression equation.

#### White blood cells count

Mice blood was collected using cardiac puncture from anesthetized mice on the eighth day after the completion of the Rane’s protocol. Mice were anaesthetized by enclosing them in a glass bottles saturated with aesthetic agent dipped in a piece of cotton wool. From the anesthetized mice blood samples were then drawn with one mL syringes moistened with heparin. Blood was packed into MiniCollect (Potassium ethylenediamine tetra acetic acid) K3 EDTA tubes (Greiner Bio One International GmbH, Kremsmunster, Austria). Samples were then taken to Muhimbili University of Health and Allied Sciences (MUHAS) clinical research laboratory for analysis by an automated hematology analyzer XS (Sysmex Corporation, Kobe, Japan). To analyses mice’s blood samples (non-human blood samples), the machine was operated on a quality control (QC) mode. The procedure (Automated hemalyzer) was used for mice of the parasite-negative control, A/LU alone and combination groups. It provided all hematological results for those groups.

#### Packed cell volume (PCV) measurement

This was done in the three groups; parasite-negative control, infected negative control and CILI extract groups. As this method uses only a small amount of blood, mice stay alive for further observation and investigation. To demonstrate the effect of the lemon fruit decoction on hemolysis, blood samples were taken from the tails of the mice and then packed in Na-heparinized capillary tubes 75 mm (Vitrex Medical A/S, Herlev, Denmark). The capillary tubes were filled to 75% full and then wax-sealed at the dry end. The tubes were placed in a Microhaematocrit centrifuge HAEMATOKRIT 210 (Andreas Hettich GmBH & Co., Tuttlingen, German) and centrifuged for 5 min at 10,000 rpm. The PCV was determined by a Standard Micro-Hematocrit reader and a ruler. Results were then calculated as a percentage of volume (height) of blood occupied by RBC divided by total blood volume (height).

#### Parasite clearance rate

The parasite clearance rate was determined by plotting the graph of average parasitemia against time for each group, and then the slope was used to evaluate clearance rates. A more robust estimation of parameters of rates of parasite clearances and lag phase were done using the Worldwide Antimalarial Resistance Network’s (WWARN’s) parasite clearance estimator (PCE) [20]. The percentage of parasitemia was multiplied by the mean number of RBC (802 × 10^6^/μL), as determined by the automated hemalyzer. The values were then submitted to the PCE online tool at the WWARN network. The student’s *t*-test then analysed the results (of A/LU alone and a combination of A/LU + CILI extract).

#### Data analysis

Data were expressed as the mean ± standard deviation (SD). Analysis of data was done using Windows statistical package for social sciences (SPSS) Version 20 (SPSS Inc., Chicago, IL, USA). One-Way analysis of variance (ANOVA) followed by Tukey’s post hoc test was performed. For each hematological parameter, the test was done in the five groups for parasitemia, the three groups of A/LU, combination and parasite-negative control. Values of *p* < 0.05 were considered statistically significant. Rates of parasite clearance were obtained from graphs plotted with the aid of Microsoft Excel 2013 (Microsoft Corp. Redmond, Washington, USA) packages. The statistical models used to estimate the parasite clearance measures and lag phase duration were fitted using the Parasite Clearance Estimator (PCE) developed by the World Wide Antimalarial Resistance Network (WWARN) [Clinical Module, WWARN. 2011 Parasite Clearance Report]. Then student’s *t-*test used to compare the rate parameters data obtained from the online WWARN’s PCE, variances were not assumed.

## Results

### Effect of CILI decoction and CILI/a/LU combination on parasitemia

Parasitemia levels taken after 72 h post-infection revealed a statistically significant difference between groups as per the one-way ANOVA (F (4,20) = 26.542, *p* = .000). A Tukey post hoc test revealed that mean parasitemia was statistically significantly lower for the A/LU (0.0 ± 0%) group compared to both the infected negative group (40.0 ± 14.78%, p = .000) and the CILI extract group (24.2 ± 9.83%, *p* = .001).

The mean parasitemia of the CILI extract group (24.2 ± 9.83%) was also lower compared to the infected negative group (40.0 ± 14.78%) *p* = 0.037. The combination group had scanty levels of parasitemia contributed by one mouse (0.6 ± 1.342). The same mouse was the one that started with the highest parasitemia of all (27%). This level of parasitemia in the combination group, however, was lower compared to both the infected negative group (*p* = .000) and the CILI group (*p* = .001). But this was not statistically significant compared to A/LU group (*p* = 1.00) and parasite-negative control group (p = 1.00).

**Fig. 1:**
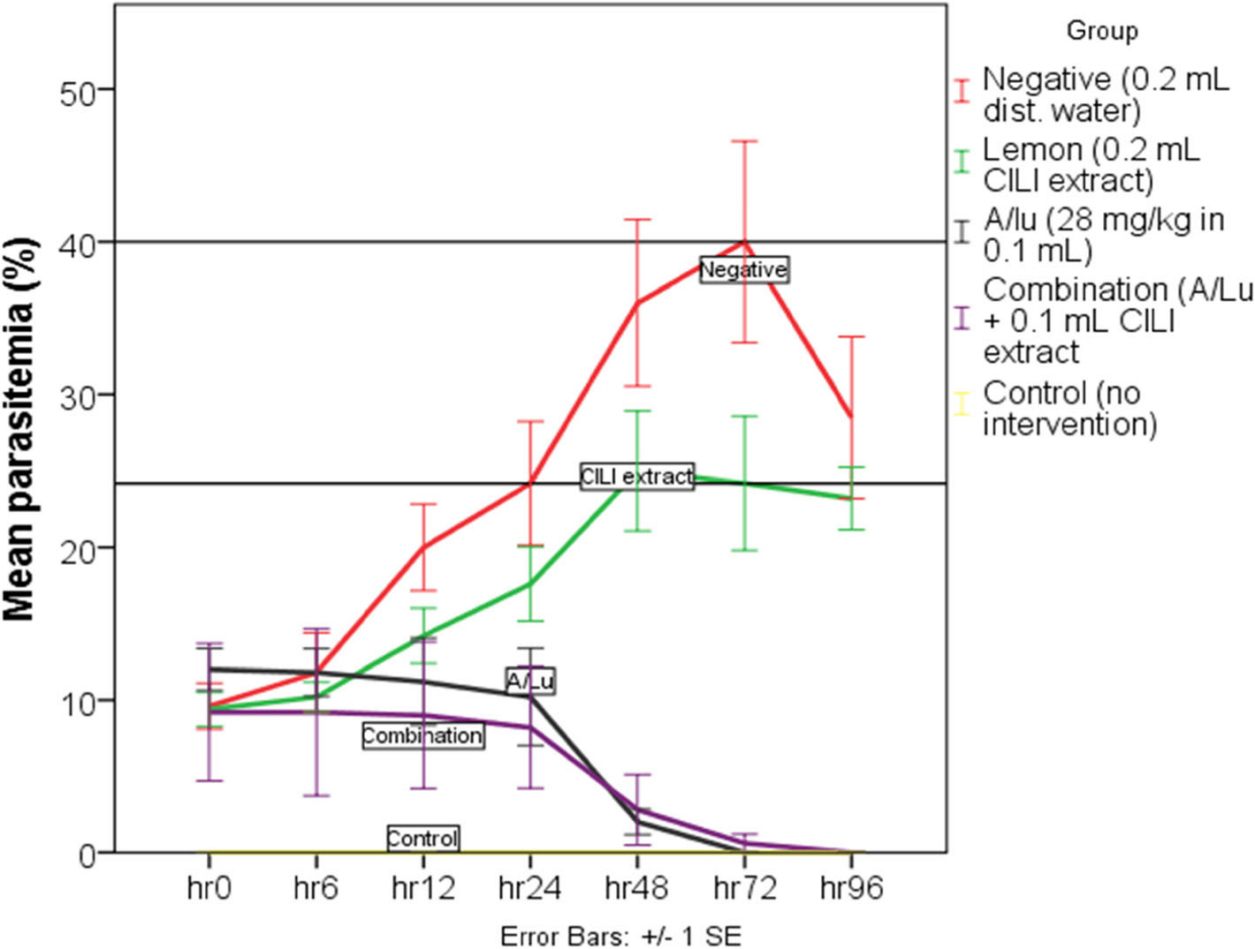
Mean percentage parasitemia in mice pre- and post-treatment

Ninety-six (96) hours post-infection all groups with standard treatment were completely cured. However, mice in the negative infected group started to die. Therefore, the parasitemia levels at 72 h were used for comparison and further analysis. The non-infected parasite-negative group (experimental control) never caught an infection in all study periods; hence, no cross-infection occurred. All parasitemia results are shown (Table 1 and Fig. 1).

**Table 1:** Mean parasitaemia ± standard deviation in *P. berghei* infected mice

Group	0 h	6 h	12 h	24 h	48 h	72 h	96 h
Negative infected	9.6 ± 3.3	11.8 ± 5.8	20 ± 6	24.2 ± 9	36 ± 12.2	40 ± 14.8	28.5 ± 13.7
0.2 ml CILI extract	9.4 ± 2.5	10.2 ± 2.2	14.2 ± 4	17.6 ± 5	25 ± 8.8	24.2 ± 9.8	23.2 ± 4.5
28 mg/kg A/LU	12 ± 3.1	11.8 ± 3.5	11.2 ± 6.4	10.2 ± 7	2 ± 1.87	0 ± 0	0 ± 0
Combination	9.2 ± 10	9.2 ± 12	9 ± 10.7	8.2 ± 8.9	2.8 ± 5.2	0.6 ± 1.34	0 ± 0
Parasite-negative control	0 ± 0	0 ± 0	0 ± 0	0 ± 0	0 ± 0	0 ± 0	0 ± 0

### Sure cure potential (SCP) of CILI decoction

**Fig. 2:**
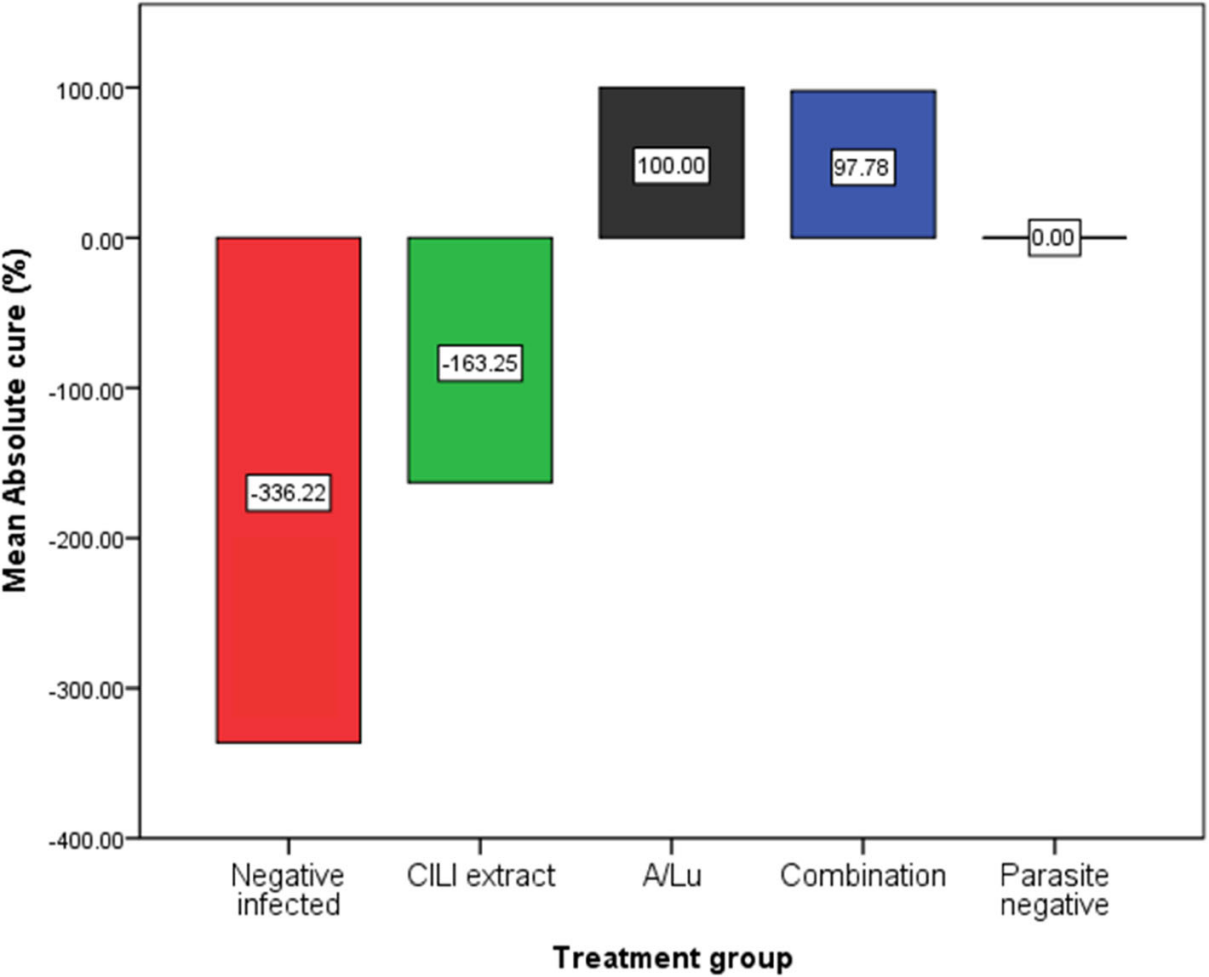
The sure curative potential of the treatments in a graphical presentation shows absolute anti-plasmodial effects of the treatments

Anti-plasmodial effect of the lemon decoction, the sure cure potential (SCP) of the decoction; using eq. 3 described in methods section these were the results; − 336.22 ± 174.54%, − 163.25 ± 100.7% for negative infected and CILI extract respectively and 100 ± 0%, 97 ± 4.97% for A/LU alone and combination treatments respectively (Fig. 2). This would suggest that on the one hand, negative infected and CILI extract groups’ parasitemia were increased the initial levels. On the other hand, the parasitemia levels of those groups with standard treatment were completely cured.

### Effect of CILI decoction on hematocrit

Hematocrit was determined using two methods; spun hematocrit procedure and automated hemalyzer method. Hematocrit by spun hematocrit procedure.

**Fig. 3:**
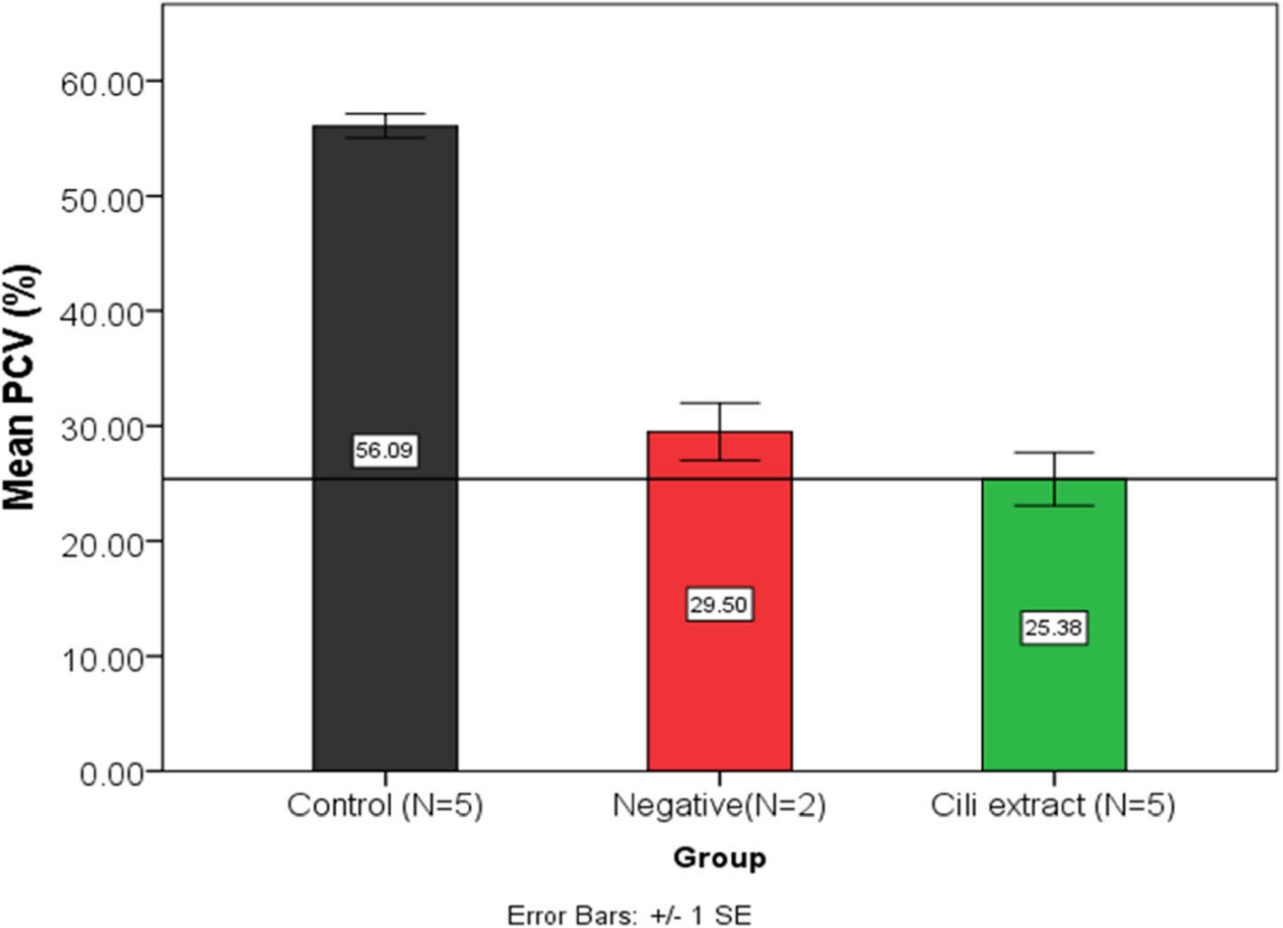
PCV (%hematocrit) in the parasite-negative (control) group (no intervention) negative infected control group (0.2 mL distilled water) and the group that received lemon decoction (0.2 mL CILI extract) determined by spun hematocrit procedure

Spun hematocrit was performed for negative infected, CILI extract and the parasite-negative control group as determined by one-way ANOVA F (2, 9) = 82.562, *p* = 0.0001). The parasite-negative (control) group had a significantly higher hematocrit (56.092 ± 2.29%) compared to the two groups, CILI extract (*N* = 5) (25.38 ± 5.158%) (*p* = 0.001) and negative infected control (*N* = 2) (29.50 ± 3.536%) (p = 0.001). The CILI extract group (N = 5) (25.38 ± 5.158%) had lower hematocrit compared to the negative infected group (N = 2) (29.50 ± 3.536%), but the difference was not statistically significant (*p* = 0.456) as shown in (Fig. 3).

### Hematocrit by automated hemalyzer method

Automated determination of hematocrit was performed for A/LU, combination (A/LU and CILI extract) and the parasite-negative control group using cardiac blood sample (terminal procedure).

**Fig. 4:**
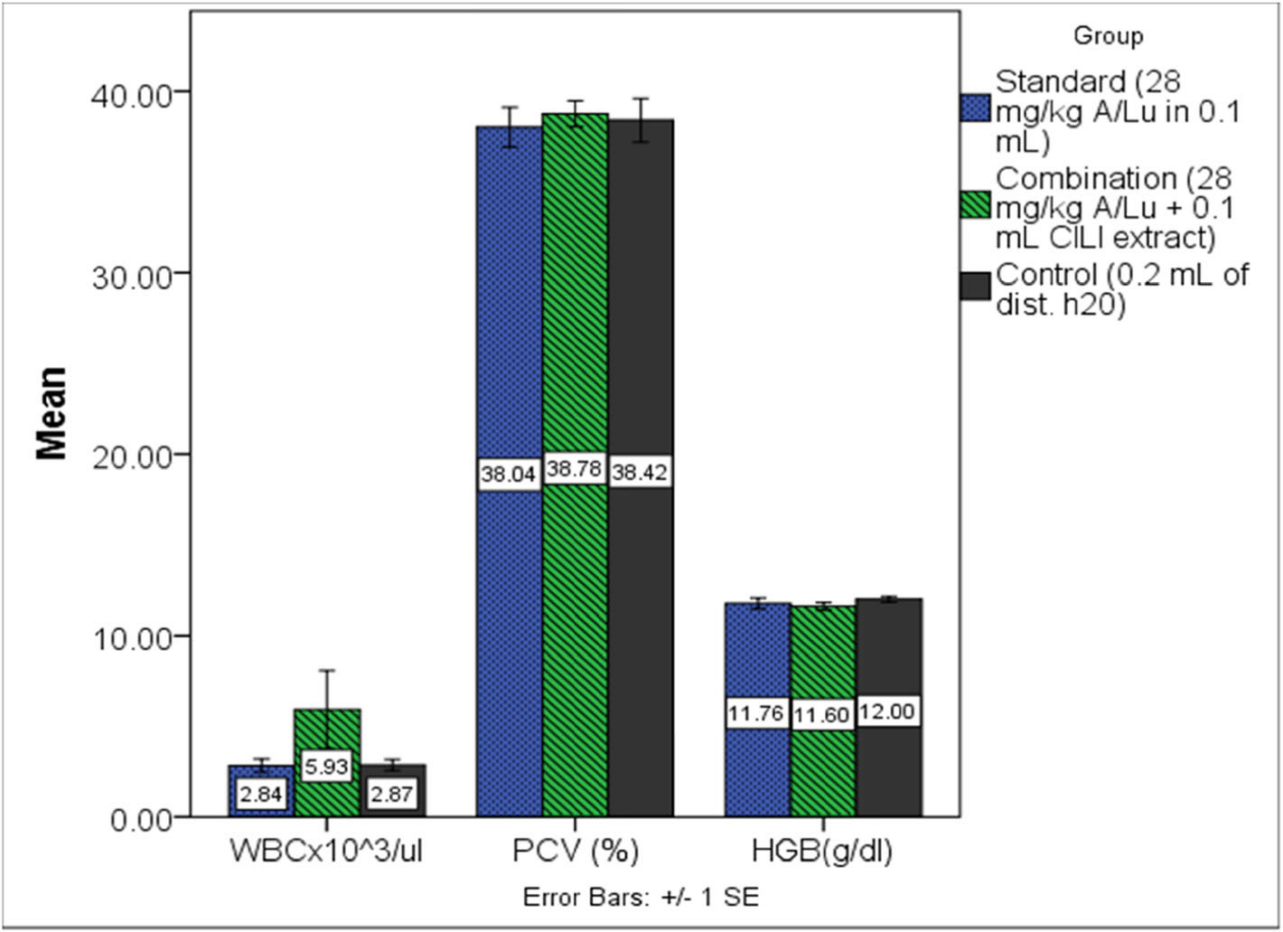
Hematological parameters of mice (5 mice in each group) when using the standard drug (A/LU) alone and when a combination of lemon decoction and standard drug (CILI extract + A/LU) were used, determined by an automated hemalyzer

As determined by One-way ANOVA F (2, 12) = 0.131, *p* = 0.879). No significant difference was obtained among means of PCV between those groups. The combination group had a hematocrit of (38.78 ± 1.594%). The A/LU group had a hematocrit of (38.04 ± 2.437) and the parasite-negative control group had a hematocrit of (38.42 ± 2.69%) as presented (Fig. 4).

### Rates of parasite clearance

From excel plots of parasite clearance taken from 0 h, 6 h, 12 h, 24 h, 48 h, 72 h and 96 h. The rate of increase in parasitemia was twice as fast in the negative infected control group (y = 6.5371x + 0.72, R^2^ = 0.9696). Compared to CILI extract group (y = 3.48x + 4.5867, R^2^ = 0.9307). The rate of parasite clearance were slightly higher in the group that received standard drug (A/LU) alone (y = − 2.4571x + 16.571, R^2^ = 0.8518) as compared to the group that received standard treatment in combination with lemon decoction (y = − 1.8214x + 12.857) R^2^ = 0.8567. Further investigation of the effect of a combination of lemon decoction and standard treatment on rates of parasite clearance, using the online platform (WWARN’s PCE) to determine the lag phase, estimated time required to clear 99% of parasites and rates of parasite clearance indicated that there was decreased lag time and time required to clear 99% of parasites in combination group (Table 2).

**Table 2:** Clearance rate parameters determined by WWARN’s Parasite clearance estimator

Measure	Group (N = 5)	Mean ± SD	t	*P*
Lag time (hour)	A/LU (28 mg/kg A/LU)	14.4 ± 10.04	1.78	0.117
	Combination	4.8 ± 6.57		
Clearance rate constant / hour	A/LU (28 mg/kg A/LU)	0.109 ± 0.03	0.084	0.936
	Combination	0.107 ± 0.02		
Intercept lag time (hour)	A/LU (28 mg/kg A/LU)	14.18 ± 0.75	−0.076	0.942
	Combination	14.23 ± 1.2		
Slope half-life (hour)	A/LU (28 mg/kg A/LU)	6.88 ± 2.25	−0.244	0.815
	Combination	6.61 ± 1.17		
R2	A/LU (28 mg/kg A/LU)	−12.9 ± 23.73	−0.649	0.55
	Combination	−5.92 ± 3.8		
Est. time to clear 50% (hour)	A/LU (28 mg/kg A/LU)	41.3 ± 16.91	0.619	0.556
	Combination	36.8 ± 10.22		
Est. time to clear 99% (hour)	A/LU (28 mg/kg A/LU)	64.2 ± 24.37	0.43	0.681
	Combination	58.8 ± 14.08		

### Effect of CILI decoction on white blood cell counts

Automated determination of WBC was performed for A/LU, combination and the parasite-negative control group using a cardiac blood sample (a terminal procedure). The results were assessed using by one way ANOVA F (2, 12) = 1.947, *p* = 0.185). The combination group had the best mean WBC count (5.934 ± 4.823%). The A/LU group had a WBC count of (2.838 ± 0.809) and the parasite-negative control group had a WBC count of (2.868 ± 0.684%). The significance values between combination vs. A/LU and combination vs. control are; *p* = 0.239 and *p* = 0.245 respectively. While control and A/LU had no significant difference, *p* = 1 (Tables 3 & 4).

**Table 3:** Antimalarial efficacy of treatments as compared to the negative infected control

Time	Negative (0.2 mL dist. water)	CILI extract (0.2 ml)	A/LU (28 mg/kg	Combination (A/LU + CILI extract)	Control
6 h	0	13.6	0	22	–
12 h	0	29	44	55	–
24 h	0	27.3	57.9	66.1	–
48 h	0	30.6	94.4	92.2	–
72 h	0	39.5%	100%	98.5%	–
96 h	0	18.6	100	100%	–

**Table 4:** Hematological parameters of mice as determined by automated hemalyzer

A/LU (28 mg/kg A/LU)	2.838 ± 0.808	38.04 ± 2.44	11.76 ± 0.65	953 ± 105.8	7.86 ± 0.33	29.9 ± 1.63	21.3 ± 1.7
Combination (28 mg/kg A/LU + 0.1 ml CILI extract)	5.934 ± 4.822	38.78 ± 1.594	11.6 ± 0.47	856 ± 138.18	8.1 ± 0.38	30.5 ± 0.85	22 ± 2.86
Control	2.868 ± 0.684	38.42 ± 2.69	12 ± 0.35	1194 ± 155	8.09 ± 0.11	29.5 ± 0.18	22.5 ± 0.8
*P*-value	0.185	0.879	0.482	0.005	0.333	0.465	0.611

### Effect of CILI decoction and CILI/a/LU combination on mean body weight, hematological parameters and survival time

The survival days of treated mice (11 ± 1.58 days) were higher compared to the survival of the negative infected group (8.6 ± 3.4 days, *p* = 0.226). On hematological parameters (Table 4), platelet count levels were the only significantly different being lower in the group that received a combination of A/LU and CILI extract. Initial weights of mice and changes in mice weights as the experiment progresses are provided (Table 5).

**Table 5:** Mean ± standard deviation weights of mice

Group (N)	Day 3	Day 4	Day 5	Day 6	Day 7
Negative infected	27.2 ± 0.84	27.6 ± 1.52	26 ± 2.83	25.5 ± 3.42	24.33 ± 4.04
	N = 5	N = 5	N = 5	*N* = 4	*N* = 3
A/LU (5)	28.8 ± 2.17	28.4 ± 2.19	27.8 ± 2.49	26.6 ± 2.88	27.6 ± 2.51
Combination (5)	28.2 ± 2.17	28.6 ± 1.95	29.2 ± 1.79	27.8 ± 1.48	28.4 ± 1.67
Control (5)	27 ± 1.22	27.4 ± 1.67	27.6 ± 2.41	27.2 ± 2.28	27.2 ± 1.92

## Discussion

Parasite suppression and antimalarial effect of the lemon decoction as evaluated using Rane’s curative test employing *Plasmodium berghei* parasites. Is an good model for studying pathological features associated with malaria infections as well as the primary screening for natural products with antimalarial activity [21]. In this study lemon decoction (CILI extract) has shown some antimalarial effect by suppressing parasite growth as depicted in Table 3. Maximum suppression of parasite growth was obtained 72 h, post- treatment. However measurements taken 96 h were considered are unreliable as already one mouse had died within the negative infected control group.

Moreover as parasitemia levels had already started to drop with and without treatment, a phenomenon that may be explained by considering sequestration of parasitized RBC in blood vessels as the infection gets severe leading to lower readings in the peripheral smears [22]. Suppression of parasitemia was 39.5% for the lemon decoction, this supports that lemon decoction may have some antimalarial activity. This observed antimalarial activity may be due to the action of various biomolecules like phenolics and minerals in lemon fruit and peel. The extraction of those biomolecules was enhanced by boiling since boiling was found to be nearly as effective as methanol in extraction in other studies [23]. We therefore suggest that future studies,further modification of dosage and methods of extraction should be implemented to assess if the activity can be increased .

Nevertheless the low antimalarial activity seen may not only due to poor extraction processes, but may be due to other factors such as lack of appropriate animal model. It well established that artificially induced infection of *P. berghei* parasites does not fully mimic the nature of malaria infections in the populations where malaria is endemic. The animal model used in this study treats the disease as an epidemic while in fact the disease is endemic in populations using this herbal remedy. These populations are endowed with immunity that has been built over a lifetime exposure to malaria infections. This this non alignment highlights the challenge of extrapolating animal test results directly to human disease [24]. In addition to this the herbal preparations might seem to work well in traditional treatment due to the additive effect of feeds. As it has been reported that in folk practices, the lemon decoction is not used alone, additives are often include; such a mix of lemons with honey lemons with sugarcane pieces, lemons with guava leaves, lemons with cinnamon and garlic [17]. It can be postulated as serendipitously combining seemingly food substances that could have resulted in something that is ‘poisonous’ to the malaria parasites. That calls for studies to be conducted with alteration of the concentration of the decoction to obtain the safe (toxicity studies) and efficacious dose or employing other methods of extraction, combining different concoctions (which was not done in this study) to determine the additive effect of other feeds in the antimalarial activity of the lemon decoction.

Anti-plasmodial effect of the lemon decoction, the sure cure potential (SCP) of the decoction. From applied mathematical calculations, substantial evidence to support direct anti-plasmodial activity is lacking (strong evidence starts from ≥0 to 100% SCP). The anti-plasmodial effect, if any, can only be speculated by considering (and under an assumption that) the rate of multiplication of *P. berghei* was higher than the rate of being killed by the lemon decoction. That little activity would then be accounted for by various biomolecules like terpenes found in lemons essential oils [25]. However, it is more evidence that there was no significant anti-plasmodial effect (at the concentration of lemon decoction used in this study). Still, only suppression of parasite growth (antimalarial activity), the antimalarial activity is to be explained by other modes of action after taking into consideration the effect of the lemon decoction on rates of parasite clearance rates and other hemato-immunological parameters of standard treated mice and mice that used a combination of treatment by the findings and in the next paragraphs.

During the course of malaria, infection anemia is brought about by mechanism yet to be fully elucidated. However, increased RBC destruction, by both splenic clearance and sequestration of damaged iRBC in blood vessels are some of those mechanisms. The lemon decoction (CILI extract) used alone did not protect mice from anemia. By observing the means of negative infected control and the group that used lemon decoction. These were both lower than the parasite-negative control group means hematocrit. The group that used lemon decoction had the lowest means. This can be explained in two ways. Firstly, that the mice that had the lowest levels of hematocrit were already dead in the negative infected group, leaving those who are having some higher levels of hematocrit skewing the results. Only three mice were alive at that time. Secondly, the lemon may be enhancing the clearance of infected RBC as part of its mode of action.

Clearance of iRBC is currently a useful marker of treatment success in malaria; however, not without its challenges. The biggest problem is that clearance rates are more of the measure of immunity than drug effectiveness. One review concluded that late parasite clearance that is also associated with treatment failure are all predicated by the same factor; host immunity [26]. The decrease in hematocrit also provides a clue in understanding the claims that lemons cause anemia that is prevalent in society. A possible explanation of that phenomenon could be that; use of lemons suppresses both fever and parasitemia in malaria-infected individuals. However, clearance of infected RBC is enhanced through immune-modulated mechanisms involving splenic destruction of RBC [27]. Since no complete cure is effected or, as a result of frequent infections, the person may suffer anemia necessitating sudden hospitalization with a significant complaint of anemia plus ‘a little’ malaria. Thus, anemia from excessive lemon users is grounded on malaria infections, fever suppression and enhanced clearance of infected RBC. Nevertheless, the advantages of low RBC and iron levels have been highlighted in other studies that it is protective of children in malaria-endemic regions [15]. Could this account for the use of lemons as prevention of malaria?

Further studies on the phenomenon of persons using lemons to stay with some levels of parasitemia without getting a fever but still suffer from anemia caused by malaria are open towards a better understanding of malaria ‘disease immunity’. This field is not well explored to date.

The results of the hematocrit of the groups that had standard treatment did not have significant differences among the three groups (*p* = 0.879). This indicates that lemons without active malaria infections do not result in RBC hemolysis. It may have had a slightly positive impact on hematology when coupled with standard antimalarial treatments.

The rates of parasite growth were twice as fast in the group that received no treatment as compared to the group that was treated with the lemon decoction. Thus lemons decrease the rate of parasite growth (suppression/inhibition). When groups that received standard treatments were compared and the rates of parasite clearance were insignificantly faster in the A/LU only group. The difference in levels of parasitemia that mice had at the beginning may have contributed to these findings. But rates of clearance are not the only determinants of how fast the treatment works in vivo.

Further investigations revealed a decreased lag time when the lemon combination of lemon decoction and standard treatment was used together. When both effects were taken into account, estimation of the time required to clear 99% of malaria parasites was lower in combination group as compared to the standard treatment alone contributed mainly by the decreased lag time. All results of rates parasite clearance were not statistically significant as shown in Table 2. The negative sure cure potential of the decoction is evidence that any added anti-plasmodial effect cannot explain the decreased lag time by combining A/LU (100% SCP) and lemon decoction (− 157% SCP). The artemether in A/LU rapidly kills malaria parasites provided it is in effective concentration in the blood regardless of immune status. The difference in outcomes was then highly contributed by the rate of clearance of those infected RBC with either dead or living parasites [26]. The use of lemon decoction possibly has enhanced immune-modulated mechanisms involving the splenic clearance of infected RBC. This is consistent with results that showed more decreased hematocrit in the group that used lemon only was less even compared to negative infected control mice provides evidence of increased clearance of infected (and non-infected) RBC. The findings are on clearance rates are also consistent with the results of a study that concluded early and faster clearance of standard treatment when used alone and when used with lime juice (*Citrus aurantifolia*) [13].

The immune-modulating potential of the decoction was performed by comparing hematological results of the standard treated mice with the control. It has been reported in studies that for an effective cure of malaria, the immunity of a person is an important factor [7]. Assessment of total white blood cell counts has shown elevated levels to a group that used a combination (A/LU + CILI extract). This signals that the decoction has immune-modulating potential. Results suggest that lemon decoction had a positive impact on the immune response of the treated mice as the combination group had the best mean counts. It must also be emphasized that this difference was not statistically significant partly because a few animals were used. Since other studies that involved rabbits reported substantial results [14]. Assessing WBC counts alone does not provide this study with enough evidence to comment that the lemon decoction had increased immunity specifically to malaria. To have that jurisdiction requires studying specific types of T and B lymphocytes and particular antibodies for malaria.

Nevertheless, the clearance of infected RBC can still be effected without specificity to malaria as it is a physiological process to clear dead, deformed and infected RBC mediated by immune-modulated splenic destruction [27]. Results of WBC are provided in Table 4. For the four days of treatment, the lemon decoction has shown some impact on malarial suppression but an insignificant impact on most hematological parameters in mice. Except platelet counts, when lemon was used together with standard antimalarial (A/LU) the platelet count was significantly lower than the control group. As Table 4 indicates, there was a significant difference in platelet counts of malaria-infected mice. The lowest value being in the combination group (the group which used A/LU and CILI extract). This feature is common as other pieces of literature have reported that malaria infections are associated with low platelet counts [28].

## Conclusion

Lemon decoction has some antimalarial activity shown by parasite suppression 39% as compared to negative infected control. Lemons used alone do not suffice as a cure but instead seems to enhance anemia associated with malaria infections in mice. However, it can be used with standard antimalarial for early parasite clearance and health outcomes on the hematology of mice. Further investigations are needed to establish the usefulness of lemons to treat/supplement treatment in endemic malaria regions. Also, new research is needed on effective methods of extraction/concentration, a proper dosage, and combinations that can achieve a cure without ill side-effects (toxicity) to the users.

## Data Availability

All data generated or analyzed during this study will be available from the corresponding author on reasonable request.

## Abbreviations

A/LU: Artemether/lumefantrine
ACT: Artemisinin-based combination therapy
BID: Twice in a day
CILI: *Citrus lemon* extract
CMC: Carboxymethyl cellulose
IPT: Intermittent Prophylaxis Therapy
iRBC: infected red blood cells
PCV: Packed cell volume
RBC: Red blood cells,
SCP: Sure Cure Potential
SD: Standard Deviations
SP: Sulfadoxine/pyrimethamine
TID: Thrice in a day
WBC: White blood cells
WHO: World Health Organization
WWARN’s PCE: Worldwide Antimalarial Resistance Network’s Parasite Clearance Estimator
